# Genomics and Epigenetics of Malignant Mesothelioma

**DOI:** 10.3390/ht7030020

**Published:** 2018-07-27

**Authors:** Adam P. Sage, Victor D. Martinez, Brenda C. Minatel, Michelle E. Pewarchuk, Erin A. Marshall, Gavin M. MacAulay, Roland Hubaux, Dustin D. Pearson, Aaron A. Goodarzi, Graham Dellaire, Wan L. Lam

**Affiliations:** 1 Department of Integrative Oncology, British Columbia Cancer Research Centre, Vancouver, BC V5Z 1L3, Canada; asage@bccrc.ca (A.P.S.); bminatel@bccrc.ca (B.C.M.); mpewarchuk@bccrc.ca (M.E.P.); emarshall@bccrc.ca (E.A.M.); gmacaulay@bccrc.ca (G.M.M.); rhubaux@bccrc.ca (R.H.); wanlam@bccrc.ca (W.L.L.); 2 Canadian Environmental Exposures in Cancer (CE2C) Network, Dalhousie University, P.O. BOX 15000, Halifax, NS B3H 4R2, Canada; a.goodarzi@ucalgary.ca (A.A.G.); dellaire@dal.ca (G.D.); 3 Robson DNA Science Centre, Arnie Charbonneau Cancer Institute, Departments of Biochemistry & Molecular Biology and Oncology, Cumming School of Medicine, University of Calgary, Calgary, AB T2N 4N1, Canada; ddpearso@ucalgary.ca; 4 Departments of Pathology and Biochemistry & Molecular Biology, Dalhousie University, Halifax, NS B3H 4R2, Canada

**Keywords:** mesothelioma, asbestos, genomics, epigenetics, non-coding RNA

## Abstract

Malignant mesothelioma is an aggressive and lethal asbestos-related disease. Diagnosis of malignant mesothelioma is particularly challenging and is further complicated by the lack of disease subtype-specific markers. As a result, it is especially difficult to distinguish malignant mesothelioma from benign reactive mesothelial proliferations or reactive fibrosis. Additionally, mesothelioma diagnoses can be confounded by other anatomically related tumors that can invade the pleural or peritoneal cavities, collectively resulting in delayed diagnoses and greatly affecting patient management. High-throughput analyses have uncovered key genomic and epigenomic alterations driving malignant mesothelioma. These molecular features have the potential to better our understanding of malignant mesothelioma biology as well as to improve disease diagnosis and patient prognosis. Genomic approaches have been instrumental in identifying molecular events frequently occurring in mesothelioma. As such, we review the discoveries made using high-throughput technologies, including novel insights obtained from the analysis of the non-coding transcriptome, and the clinical potential of these genetic and epigenetic findings in mesothelioma. Furthermore, we aim to highlight the potential of these technologies in the future clinical applications of the novel molecular features in malignant mesothelioma.

## 1. Introduction

Malignant mesothelioma is an aggressive and highly fatal cancer associated with exposure to asbestos [1,2]. It is characterized by a long and remarkably variable latency period between exposure and disease presentation (13–70 years) and a poor survival rate, wherein most patients will succumb to the disease within the first year after diagnosis [3,4,5]. Despite global efforts to limit asbestos exposure through bans and mine closures in numerous countries, a corresponding decrease in mesothelioma incidence has not been observed. In fact, the incidence of mesothelioma increased by almost 40% between 2005–2015 [6,7,8]. Further, this increase can be attributed to factors beyond persistent asbestos exposure, including the environmental exposure to other mesothelioma-inducing mineral fibers such as erionite, carbon nanotubes, and fluoro-edenite, as well as the carcinogenic side effects of radiation used in the treatment of other cancers [9].

The mesothelial cells of the pleura that lines the lungs are the most frequent site of mesothelioma development; malignant pleural mesothelioma (MPM) accounts for 70–80% of all cases. However, other serosal membranes, such as the peritoneum (peritoneal mesothelioma (PeM); ~25% of cases), as well as the pericardium and the tunica vaginalis, are also affected [10,11]. Histologically, mesothelioma can be classified into three variants: (i) epithelioid, (ii) sarcomatoid, and (iii) mixed/biphasic [11,12]. While immunohistochemical (IHC) markers have been improved in recent years, the lack of sensitive and specific disease subtype-specific markers continues to hamper diagnosis of malignant mesothelioma [10,13]. Specifically, even when abnormal mesothelial proliferations are identified by IHC in serosal membranes, it is difficult to distinguish benign from malignant growths. Currently, diagnosis relies heavily on morphology, where malignant growths are characterized by deep stromal invasion with dense cells and complex growth patterns [11]. Further, if the proliferations are confirmed as the epithelioid subtype of malignant mesothelioma, it is critical to consider the potential invasion of carcinomas from other tissues, especially the lung, breast, and ovary [11,13,14,15]. Alternatively, sarcomatoid mesothelioma proliferations warrant the exploration of confounding sarcomatoid malignancies [11].

Asbestos fibers have been classified as human carcinogens by the International Agency for Research on Cancer (IARC) [16]. These fibers have been used commercially for decades; while yearly cases now extend into the thousands, malignant mesothelioma was practically absent in the 1950s [7]. Asbestos-related risks have mainly been deduced from cases occurring in mine workers and their close relatives [17,18,19]. Yet, a significant proportion of the newly diagnosed cases of malignant mesothelioma are the result of other (non-mining) professional occupations, as well as environmental exposure [20,21,22]. Together, these data indicate that the current and future burden of mesothelioma is largely underestimated. 

Over the last few years, an increasing number of studies have comprehensively characterized different omics dimensions of mesothelioma. In this article, we review the advances in molecular and clinical aspects of mesothelioma obtained by the characterization of the genomic and epigenetic landscape of mesothelioma using diverse high-throughput technologies. Collectively, we discuss how these molecular features may direct novel clinical applications in the treatment of mesothelioma.

## 2. Molecular Mechanisms of Asbestos-Related Carcinogenesis

**Figure 1:**
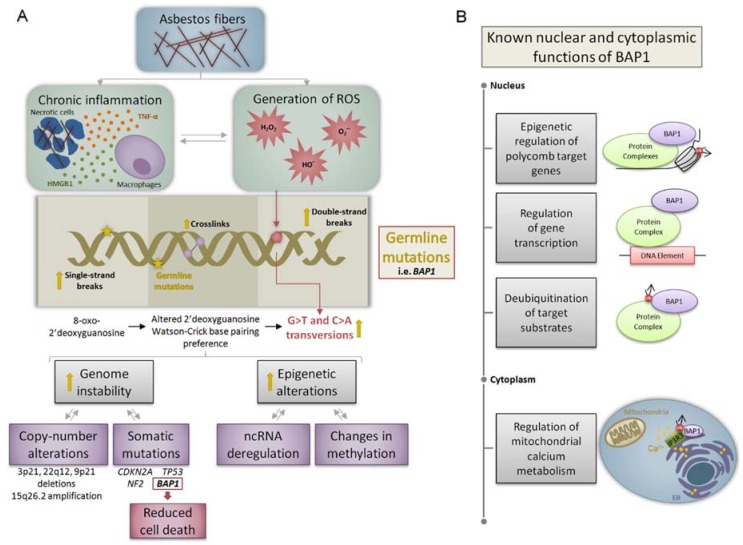
Molecular outcomes of exposure to asbestos fibers. (**A**) Asbestos-related carcinogenic effects mainly occur through two mechanisms: activation of chronic inflammation and generation of reactive oxygen species (ROS). Both mechanisms are known to promote DNA damage in the forms of single-strand breaks, crosslinks, and double-strand breaks. Particularly, the oxidation of the 8th carbon on the DNA base guanine (8-oxo-2′deoxyguanosine, red pentagon) changes normal 2′deoxyguanosine Watson–Crick base pairing preference from 2′deoxycytosine to 2′deoxyadenosine, resulting in G to T and C to A transversions. The final outcome of the oxidative DNA damage is the triggering of genomic stability and numerous epigenetic alterations. Finally, the impact of ROS can be exacerbated (yellow arrows) by the presence of germline mutations (yellow stars) that affect the DNA damage repair machinery of the cell. Ultimately, the deregulation of gene expression caused by these mechanisms lead to altered cellular processes, such as cell death. (**B**) BRCA1-Associated Protein 1 *(BAP1)* acquired and germline mutations are the most common alterations observed in mesothelioma, affecting gene transcription and promoting post-transcriptional modifications through ubiquitination changes (red circle). The most well-known functions of BAP1 occur in the nucleus, where it promotes the maintenance of genomic stability. However, BAP1 can also exert functions in the cytoplasm, where it localizes to the endoplasmic reticulum (ER) and modulates calcium (Ca^2+^) release through binding and deubiquitination of the type 3 inositol-1,4,5-trisphosphate receptor (IP3R3) [29]. The modulation of Ca^2+^ release from the ER to the cytosol and mitochondria promotes apoptosis. Therefore, reduced levels of BAP1 promote both genomic instability and reduced cell death, favouring malignant transformation. ncRNA: non-coding RNA.

As asbestos exposure is a primary cause of mesothelioma development, analysis of the molecular aberrations induced by these fibers is pertinent to the study of mesothelioma biology. The primary mechanism of asbestos-related carcinogenesis is chronic inflammation and ongoing generation of highly reactive oxygen species (ROS) that collide with cellular components, promote DNA mutation, and trigger transformation [23]. Asbestos fibers also contain iron (II) ions (Fe^2+^) and can induce hemolysis to sequester Fe(II) from hemoglobin [24]; this is particularly important as, via the Fenton reaction, free Fe(II) disproportionates H_2_O_2_ into hydroxyl radicals (^●^OH) that oxidize DNA, free nucleic acids, proteins, and lipids [25]. This process is exacerbated by the release of cytokines, including tumor necrosis factor-α (TNF-α) from macrophages and high mobility group box 1 (HMGB1) proteins from necrotic cells, amplifying the inflammatory response and increasing the number of cells undergoing oxidative damage [26]. Oxidative DNA damage, if not properly repaired, is highly mutagenic and can trigger genomic instability, a primary enabling characteristic of cancer formation that is detectable by high-throughput techniques (Figure 1a) [27].

A multitude of oxidative DNA lesions, including oxidized DNA bases, abasic sites, single-strand DNA breaks (SSBs), DNA double-strand breaks (DSBs), and DNA intra- and inter-strand crosslinks (ICLs) each require distinct DNA repair pathways to resolve. Double-strand breaks and intra- and inter-strand crosslinks are particularly toxic, as they can lead to the collapse of replication forks but are also primary drivers of chromosomal rearrangements, chromosome gain, loss and/or fragmentation [28]. Whilst the DNA damage arising from short, acute bursts of ROS are generally resolved quickly and without long term consequence, chronic ROS generation via inflammatory processes reacting to asbestos fibers produce dangerous oxidatively stressed tissue microenvironments. Within such environments, chronic oxidative stress accelerates the pace of genetic mutation, giving rise to cancer with alarming efficacy and speed.

## 3. Genomic and Epigenetic Landscape of Malignant Mesothelioma

Multi-omics studies have proven to be an insightful approach to characterizing the intricacies of tumour biology [28]. The Cancer Genome Atlas (TCGA) initiative has been a valuable resource for cancer research, focusing on multi-omic analyses of a variety of tumor types [28] (Table 1, Table 2 and Table 3). Specifically, the TCGA-MESO cohort presents 87 MPM cases with comprehensive DNA, RNA, and epigenetic profiles that are publicly available through the Genomic Data Commons portal [30].

The gene encoding BRCA1-Associated Protein 1 (BAP1) serves as the most prevalent example of the impact of these high-throughput approaches, as it has been identified as one of the most frequently altered genes in mesothelioma through multiple molecular mechanisms (Figure 1b) [46]. Moreover, the clinical potential of *BAP1* alterations is being intensively investigated (discussed below). Other frequently mutated genes in mesothelioma include *NF2*, *CUL1*, and *TP53* (Figure 1a) [36,47]. Aside from mutations affecting single genes, different mutational signatures have been identified in mesothelioma. Interestingly, these signatures are not significantly different between cases with or without known asbestos exposure. However, the mutational signatures of a subset of mesothelioma samples are consistent with base-agnostic mutagens such as ROS, which are characterized by no preference for specific types of transitions or transversions [47].

Molecular events that alter the number of DNA copies of a specific gene are common in tumorigenesis. Recent comparisons between mesothelioma and other tumors have provided some insight into the DNA-level biology of mesothelioma (Table 2). Analysis of DNA-damage-repair gene somatic alterations across TCGA tumors revealed that mesothelioma had a DNA damage repair footprint significantly associated with progression-free and overall survival [48]. Moreover, frequent DNA-level alterations—specifically copy-number losses—affecting *CDKN2A*, *NF2*, *EP300*, *SETD2*, *PBRM1*, and *BAP1* have been detected in copy-number data from TCGA and additional cohorts. These results were confirmed by low-pass whole genome sequencing in a subset of tumors [49].

**Figure 2:**
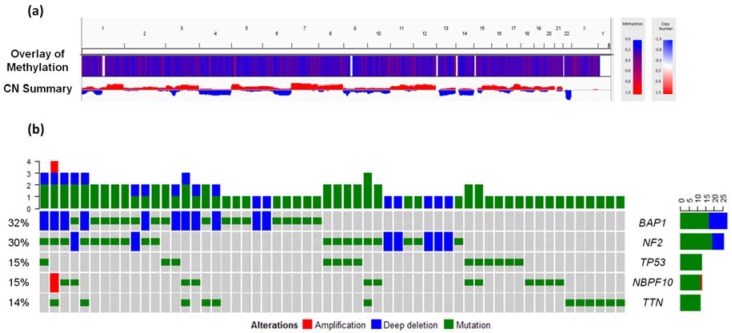
DNA level alterations in the TCGA Mesothelioma cohort. (**a**) Methylation (average methylation β-values) and Copy Number changes (CN) in 87 mesothelioma samples processed by The Cancer Genome Atlas (TCGA). Data graph was generated using the Integrative Genomics Viewer (IGV) [51,52]. (**b**) Specific gene level alterations from the 5 most frequently mutated genes *BAP1*, *NF2*, *TP53*, *NBPF10*, and *TTN*, in the same 87 mesothelioma samples. Samples that had no alterations were excluded from the visualization. The top bar graph summarizes the number of alterations per sample and the bar graphs to the right represent the number of alterations per gene. Graphs were generated using the OncoPrint function of the ComplexHeatmaps R package [53].

Whole-exome sequencing analyses have also identified frequent alterations affecting *BAP1*, *NF2*, and *CDKN2A* (located in chromosomal regions 3p21, 22q12, and 9p21) by somatic mutations and/or copy-number alterations (Figure 2b) [36]. Furthermore, a combined approach using high-density comparative genomic hybridization (CGH) and targeted next-generation sequencing (NGS) was used to explore the 3p21 region in detail. The results revealed biallelic gene inactivation of *SETD2*, *BAP1*, *PBRM1*, and *SMARCC1*, suggesting that the mesothelioma genome is affected by minute deletions that are non-detectable by singular NGS-based approaches or commercial array CGH [50].

Recently, an analysis of copy-number dosage changes in peritoneal mesothelioma cases revealed a similar genomic landscape to that described for the more commonly studied pleural mesothelioma, including the loss of chromosomal regions 3p21, 9p21, and 22q12. The authors describe two novel genomic alterations preferentially occurring in peritoneal mesothelioma (amplification of 15q26.2 and deletion of 8p11.22). However, the biological relevance of these alterations has not been established [54]. Analysis of average methylation and copy number alterations in the 87 samples of the TCGA-MESO cohort confirms these previously-described genome-wide alterations in mesothelioma, such as the prevalent copy-number loss of chromosomal region 22q12 (Figure 2a). This cohort exhibits a significant fraction of samples with *BAP1* aberrations. Furthermore, the most recent analysis of mesothelioma samples (TCGA-MPM) suggests up to 57% of samples with alterations in *BAP1*, collectively confirming that *BAP1* is the most frequently altered gene in mesothelioma [43].

Although copy number and methylation changes in mesothelioma have been characterized (Figure 2a), the epigenomic landscape of mesothelioma has been analyzed to a lesser extent than the genomic factors of this cancer. One of the first studies aiming to characterize the epigenomic landscape of mesothelioma and its biological implications was performed by Christensen et al. [55], which identified significant differences amongst the epigenetic profiles of malignant mesothelioma compared to normal pleura. Moreover, specific methylation patterns were found in patients with evidence of asbestos exposure. A more in-depth analysis of the correlation between genetic and epigenetic changes in mesothelioma identified that methylation status and copy-number were significantly associated in the *TGFB2* and *GDF10* genes. However, tumors without DNA losses affecting the region encoding for the key methyltransferase enzyme DNMT1 were found to have higher average methylation across all CpGs [56]. Indeed, recent quantitative reverse transcription polymerase chain reaction (RT-qPCR) analyses of asbestos-associated MPM cell lines identified over-expression of *DNMT1*, *DNMT3A*, and *DNMT3B*, as well as other key epigenetic regulators including *EZH2* and *SUZ12* [57]. Moreover, the high expression of these epigenetic modifiers was shown to be significantly correlated with poor survival of individuals with MPM [57]. Further, cytokine signaling—induced by proteins including high mobility group box 1 (HMGB1) and NACHT, LRR and PYD domains-containing protein 3 (NALP3; encoded by the *NLRP3* gene) (involved in the asbestos response)—can affect both *DNMT* expression and subsequent methylation of target genes [26,57,58]. Interestingly, the chromatin binding protein ASXL1, which directly interacts with enhancer of zeste homolog 2 (EZH2) and polycomb repressive complex 2 subunit (SUZ12) to direct Polycomb repressive complex 2 (PRC2) binding to DNA, also directly interacts with BAP1 [59,60]. Thus, BAP1 binding ASXL1 may act to inhibit the repressive activities of PRC2 and promote gene expression. However, as both sporadic and familial MPM has a high prevalence of inactivating *BAP1* mutations, this epigenetic axis may be critical to mesothelioma carcinogenesis [57]. Finally, *Bap1* loss in mice was shown to result in increased expression of both *Ezh2* and subsequent hypermethylation of PRC2 targets [61]. Collectively, these results suggest not only the importance of epigenetic regulation in mesothelioma biology, but also highlight the potential utility of epigenetic-based therapies such as EZH2 inhibitors. To investigate the role of epigenetic alterations contributing to tumor heterogeneity, Kim et al. [62] characterized epigenomic and transcriptomic alterations in a side population of a mesothelioma cell line (MS-1), using MeDIP-seq and RNA-seq. Epigenetic alterations and consequent transcriptomic changes in the *BNC1*, *RPS6KA3*, *TWSG1*, and *DUSP15* genes were characteristic features found in the side population cells and likely to participate in defining tumor heterogeneity.

Further, it has been shown that the type of asbestos fiber can influence the epigenetic landscape of mesothelioma cases. Mesothelial cell lines (Met5A) exposed to chrysotile exhibited altered methylation patterns in intergenic regions, while crocidolite mainly affected gene-coding regions. Interestingly, no significant correlation was observed between methylation changes and global mRNA expression levels, with the only exception being the *DKK1* gene in cells exposed to chrysotile [63].

### Genetic Variants Modifying the Risk of Malignant Mesothelioma

Germline mutations in the *BAP1* gene are one of the most significant factors that lead to the development of mesothelioma [64]. Additional findings from genome-wide association studies (GWAS) have confirmed the sensitizing effects of *BAP1* and have also expanded to other genes participating in the *BAP1* interaction network. For example, analysis of 407 pleural mesothelioma cases and 389 controls with a comprehensive history of asbestos exposure revealed an increased risk of abnormalities in chromosomal region 7p22.2, which includes the gene encoding Forkhead box protein K1 (FOXK1) that is known to interact with BAP1 [65]. However, it is critical to probe genes beyond BAP1 that confer differential susceptibility to mesothelioma development and treatment response.

Recent studies have since begun to identify additional susceptibility candidates. In Western Australia, a large study (428 cases and 1269 controls) identified variants located in the *CRTAM*, *SDK1*, and *RASGRF2* genes that were significantly associated with malignant mesothelioma risk [66]. In cases like these, it is common to focus on the interactions between exposure to a specific environmental carcinogen (e.g., asbestos) and single-nucleotide polymorphisms (SNPs) emerging from previous GWAS studies. For instance, these approaches have revealed the synergistic effect between asbestos exposure and rs1508805, rs2501618, and rs5756444 genetic variations [67].

The results obtained to date by these limited number of GWAS-based analyses only suggest that the genetic background modulates the main effect of asbestos in the carcinogenesis of the pleura, rather than directly conferring differential disease susceptibility. However, further studies of additional cohorts, as well as mechanistic studies focused on GWAS-identified candidates may elucidate the relationships between genotype, phenotype, and susceptibility to malignant mesothelioma.

## 4. Clinically Relevant Genes Identified through High-Throughput Analyses

### 4.1. BRCA1-Associated Protein 1 (BAP1)

The discovery of the role of *BAP1* mutations in malignant mesothelioma is an example of the combination between classical genetic methods and novel high-throughput technologies to discover causal relationships between genotype and phenotype. A case in point is Cappadocia, which displayed a significantly higher rate of deaths related to mesothelioma relative to neighboring regions in Turkey with high asbestos usage. This fact, together with an autosomal dominant transmission pattern is highly suggestive of genetic-based susceptibility. Screening of genealogies from affected families led to the discovery of an inherited cancer syndrome caused by *BAP1* germline mutations [68,69].

The loss of *BAP1* and *CDKN2A* are genetic events shown to effectively differentiate between malignant mesothelioma and reactive mesothelial hyperplasia [70]. However, *BAP1* mutations are also found in other types of tumors, thus its alteration is not exclusive to mesothelioma. BAP1 is a deubiquitinating enzyme which participates in DNA repair and gene expression processes [46]. Therefore, its loss likely contributes to mesothelioma development by both transcriptional mechanisms as well as increased genomic instability, a recognized hallmark of cancer development and progression (Figure 1) [27]. In fact, *BAP1* has been shown to be affected in both alleles by copy-number losses and mutations in 42% of tumors [71]. Additionally, the high frequency of mutation in BAP1, as well as *NF2* and *TP53* was confirmed in multi-omic analysis of human malignant and matched non-malignant samples [47].

Interestingly, it has been shown that germline mutations in *Bap1* can induce epigenetic deregulation of the Rb protein in mice, facilitating the development of malignant mesothelioma [72]. Moreover, mice lacking *Bap1* are sensitive to EZH2 pharmacologic inhibition, suggesting a novel epigenetically-based therapeutic approach for BAP1-mutant malignancies [61]. Additional studies have suggested that *BAP1* mutations could be further exploited for MPM epigenetic therapy, since BAP1 function stabilizes BRCA1 and promotes recruitment of the polycomb deubiquitylase complex to DNA damage sites [73,74].

### 4.2. Deletion in 9p21

Deletion of 9p21 is a common event in malignant mesothelioma. This region contains the *CDKN2A* gene, which encodes p16, a well-established tumor suppressor in a variety of tumor types, including malignant mesothelioma. Loss of the *CDKN2A* gene and loss of activity has been confirmed in several studies and has been correlated with poor patient prognosis [75,76,77]. Deletion of *CDKN2A* is commonly assessed through fluorescence in-situ hybridization (FISH), which is widely used as a molecular diagnostic tool in malignant mesothelioma. 

Deletion of *CDKN2A* occurs in up to 80% of pleural mesothelioma cases, and the frequency is higher in sarcomatoid tumors. However, this alteration is only observed in 25% of peritoneal mesothelioma cases [10]. Recent studies indicate that while a positive identification of *CDKN2A* deletion is consistent with malignant mesothelioma, the lack of deletion does not preclude disease diagnosis [78]. Additionally, deletion of *CDKN2A* is not useful in distinguishing mesothelioma from metastatic lung tumors, a common clinical problem in mesothelioma diagnosis [10,79].

### 4.3. Additional Genomic Disruptions

Initial studies using RNA pyrosequencing techniques, revealed multiple types of genetic alterations commonly occurring in malignant mesotheliomas, including somatic mutations, gene deletions, gene silencing, and RNA editing [32]. The combination of RNA-seq experiments with targeted exon sequencing have since unveiled other disruptions that may be critical to mesothelioma development in some patients. Namely, it has been observed that the Hippo pathway—involved in the definition of organ size through the regulation of cell proliferation and apoptosis—is amongst the most frequently inactivated in mesothelioma. Several genes encoding for members of this pathway, including *NF2*, *RASSF1/2/6*, *LATS1/2*, and *FAT1* were affected by mutations, copy number changes or loss of expression [80]. Moreover, YAP1—a downstream effector of the Hippo signaling pathway—is also activated in mesothelioma as a result of DNA amplifications and has been proposed as one of the few clinically actionable options in mesothelioma [80,81]. Additionally, analysis of the newly released TCGA-MPM cohort revealed focal deletions in numerous genes, including *BAP1*, *SETD2*, *PBRM1*, *LATS1*, *PTPRD*, *CDKN2A*, and *MTAP*, as well as a lack of genomic disruptions involving targetable genes and signaling pathways such as MAPK and PI3K/AKT [43]. 

High-throughput analyses have revealed other single gene disruptions in mesothelioma samples. For instance, the *MTAP* gene is also located in the 9p21 region that is commonly deleted in malignant mesothelioma. Recent studies have investigated the use of methylthioadenosine phosphorylase (MTAP) IHC in combination with BAP1 to distinguish benign from malignant mesothelial proliferations, particularly from pleural effusion samples [82]. While MTAP IHC alone showed low sensitivity (42.2%), the combination with BAP1 IHC increased this parameter up to 77.8%. These findings are particularly relevant in clinics where the use of FISH techniques (used for p16 assessment) is not a routine practice or where the appropriate infrastructure is not available.

Similarly, *NF2* (located in 22q12) encodes the Merlin protein, which functions as a tumor suppressor. It is inactivated by mutation in ~40% of malignant mesothelioma, and this loss of function promotes invasiveness. Re-expression of Merlin inhibits a number of malignant properties such as cell motility and invasion (via regulation of focal adhesion kinase (FAK) activity) [83,84,85,86]. It has been proposed that this feature can be therapeutically exploited, since mesothelial cells can be sensitized to FAK inhibition by the loss of *NF2* [87]. However, clinical trials have only provided limited evidence of the clinical utility of treatments with FAK inhibitors.

The use of microarray technologies has resulted in the generation of multi-gene signatures associated with malignant mesothelioma patient prognosis [88,89]. For instance, de Reynies et al. used transcriptomic microarray analyses to stratify MPM patients into two subgroups based on a three-gene signature, where different molecular profiles were indicative of a differential disease subtype and survival outcome [90]. High-coverage multi-gene studies can not only confirm key molecular events described in single-gene studies, but also uncover less common features that may be driving mesothelioma development, such as the loss of tumor suppressor genes *SETD2*, *PBRM1*, and *PTEN* [47]. However, despite demonstrated accuracy of these multi-gene signatures in identifying mesothelioma within a cohort, the results have yet to be extended to other samples or cohorts [89]. Thus, further validation is required in order to confirm the prevalence of these alterations in different populations, which will aid in the translation of these findings to clinical practice.

## 5. Non-Coding Transcriptome as a Tissue-Specific Feature in Malignant Mesothelioma

Broad exploration of the genome made possible by the advent of next generation sequencing has revealed roles for RNA transcripts that do not encode proteins in the regulation of gene expression; species that are termed non-coding RNAs (ncRNAs). Categorized on the basis of size—small ncRNAs (<200 nt) and long ncRNAs (>200 nt)—these transcripts have emerged as key regulators of critical processes, such as the cell cycle, proliferation, and tumorigenesis [91,92,93]. 

### 5.1. MicroRNAs 

Perhaps the most well-studied species of the non-coding transcriptome are microRNAs (miRNAs), small (18–22 nt) transcripts that regulate mRNAs through direct base-pairing interactions of as little as six nucleotides [94]. MiRNAs have been extensively described in diverse areas of cancer biology—including mesothelioma—yet their clinical utility is still an area of active investigation. One of the first studies of miRNA dysregulation in malignant mesothelioma revealed clear differences in miRNA expression profiles between tumor samples and normal controls [95]. Some of the deregulated miRNAs identified had been previously detected as disrupted in other cancer types, affecting pathways related to cell cycle regulation, proliferation and migration; others were shown to map to genomic locations known to be deleted or gained in malignant mesothelioma [49,95,96]. Namely, miR-30b* was found to be overexpressed in MPM and locates to 8q24, a frequently gained region in mesothelioma. Likewise, miR-34* and miR-429 located at 1p36, as well as miR-203 located at 14q32, are not expressed in tumor samples and represent regions frequently affected by DNA copy-number losses [95,97] (Table 4).

Importantly, miRNA expression patterns are known to be not only tumor-type specific but also differ according to distinct tumor subtypes [119]. For example, the epithelioid subtype of malignant mesothelioma is characterized by the expression of miR-135b, miR-181a-2*, miR-499-5p, miR-517b, miR-519d, miR-615-5p, and miR-624, while the biphasic subtype expresses miR-218-2*, miR-346, miR-377*, miR-485-5p, and miR-525-3p, and miR-301b, miR-433 and miR-543 are specific to the sarcomatoid subtype [95]. The advantage of such specificity in miRNA expression patterns is that they could be explored as diagnostic and prognostic tools, particularly due to the fact that their sequence size promotes a high stability in body fluids, such as blood and urine [120]. Table 4 summarizes evidence of the ability of miRNAs to provide diagnostic and prognostic information for malignant mesothelioma patients. 

While some miRNAs such as miR-29c* and miR-625-3p are detectable in plasma and can differentiate malignant mesothelioma patients from healthy individuals [100]. Other miRNAs could be used to overcome the limitation of differentiating between mesothelioma and other malignancies that have metastasized to the pleural or peritoneal cavities. In fact, miRNA expression profiles from the miR-200 family have shown great potential in distinguishing malignant mesothelioma from lung adenocarcinomas [102,103]. Additionally, miRNAs have also been shown to predict patient outcome and response to therapy. MiR-15b, miR-16, miR-193a-3p, miR-195, and miR-200c are all associated with increased expression of programmed death-ligand 1 (PD-L1), which not only indicates a poor prognosis but could also inform the efficacy of the programmed cell death protein 1 (PD-1)/PD-L1 immunotherapy regime [114].

Although the use of miRNAs as diagnostic, prognostic and even therapeutic tools is a promising field, further studies are needed to overcome important limitations, such as sample sources and size, and the analysis of only a limited number of miRNAs. Therefore, larger sample cohorts and the combination of high-throughput technologies with other techniques such as RT-qPCR and in-situ hybridization would aid in the elucidation of the role of miRNAs in mesothelioma. Additionally, in the generation of miRNA-based signatures, it is important to highlight that the use of different platforms such as microarray, RT-qPCR, and RNA-seq may provide conflicting results. Likewise, observations made in cell lines do not always translate to clinical practice, thus the validation of these results in patient samples should be prioritized. For example, loss of miR-31 that is located at 9p21.3—a region frequently lost in MPM—has been shown to be significantly associated with poor prognosis and a short time to tumor recurrence [117]. However, despite being downregulated in malignant mesothelioma compared to benign reactive mesothelial proliferations, Matsumoto et al. demonstrated that patients with the sarcomatoid subtype of MPM and upregulation of miR-31 have a worsened overall survival compared to sarcomatoid cases without miR-31 upregulation [116]. Thus, further studies with higher sample sizes and comparable high-throughput techniques are required to elucidate the prognostic value of miR-31 in mesothelioma management.

Recently, high-throughput studies have revealed that the current number of annotated miRNAs only represents a fraction of the total pool expressed by the human genome. These previously unannotated miRNAs have a high degree of tissue specificity and display expression patterns indicative of a role in the regulation of gene expression in a number of cancers [121,122,123,124]. Their high specificity accounts for how they have evaded detection, but more importantly, highlights their potential utility as sensitive markers of easily confounded tumor types like malignant mesothelioma. Considering the potential of these novel miRNAs as tissue-of-origin markers, they may represent ideal biomarkers for malignant mesothelioma only detectable through high-throughput RNA technologies.

### 5.2. Long Non-Coding RNAs 

As long non-coding RNAs (lncRNAs) can exert their regulatory effects at the DNA, RNA, and protein levels, and have observed tissue-specific expression patterns, they present exciting and broad opportunities for both diagnostic and prognostic markers. Thus, characterizing the landscape of lncRNA deregulation in mesothelioma has the potential to reveal novel insights into mechanisms of malignant mesothelioma-associated gene regulation as well as novel therapeutic intervention points.

While most current studies on lncRNAs focus on one lncRNA-phenotype relationship, the overall dysregulation of lncRNAs has been observed in pleural mesothelioma tumors (Table 5) [125]. In fact, a recent study showed 6 biologically-validated lncRNAs that could distinguish malignant from benign pleural tissue [126]. Beyond this, a number of specific lncRNAs have also been shown to be directly relevant to mesothelioma biology, from disease onset to subtype differentiation. Asbestos-exposed mice with and without tumor development show differential lncRNA expression patterns, of which the most significantly dysregulated is FOXF1 adjacent non-coding developmental regulatory RNA (FENDRR), which shows increased expression in epithelioid tumors [127]. Additionally, lncRNA knockdown studies have revealed a role for growth arrest-specific 5 (GAS5) in tumor cell quiescence and the modification of cell proliferation and PVT1 oncogene (PVT1) in the regulation of apoptosis and cell proliferation [128,129]. Interestingly, lncRNAs have been observed in features directly relevant to malignant mesothelioma, such as the histotype transition from epithelioid to sarcomatoid forms of pleural mesothelioma [130]. These lncRNAs may be useful as histotype markers in MPM, which would significantly aid in providing an accurate clinical diagnosis. For instance, prostate cancer associated transcript 6 (PCAT6) is observed to be significantly upregulated in the biphasic morphology of MPM, while nuclear enriched abundant transcript 1 (NEAT1)—known to promote epithelial-to-mesenchymal transition—is mainly downregulated in MPM, but a proportion of epithelioid tumors show NEAT1 overexpression [130].

Since the evidence of the involvement of lncRNAs in the development of mesothelioma has only recently emerged (Table 5), their exact diagnostic and prognostic utility remains largely unexplored in this cancer. However, as many of the current genomic aberrations that have been described in malignant mesothelioma are not clinically actionable, the non-coding transcriptome may represent an alternative method of gene regulation relevant to mesothelioma biology. Thus, the analysis of the non-coding areas of the mesothelioma genome may uncover not only new players in tumor biology but also intervention points for patients who do not harbor currently defined molecular drivers.

## 6. Conclusions and Future Challenges

Overall, mesothelioma represents an enormous burden on public health worldwide, particularly in light of the prevalence of asbestos fibers in the environment. The high latency periods characterizing mesothelioma development and the growing environmental exposure to asbestos support the hypothesis that mesothelioma disease rates will not necessarily decrease with increased implementation of strict asbestos-related sanctions. Thus, identifying and developing both high-level strategies for the management of asbestos exposure, as well as promoting whole-genome and epigenomic analysis of mesothelioma is of the utmost importance to preventing the impact of malignant mesothelioma on public health.

The use of high-throughput technologies has improved our understanding of the genomic and epigenomic factors relevant to malignant mesothelioma. These types of analyses have also revealed the role of the non-coding transcriptome, which significantly expands the spectrum of functional gene networks that may be involved in mesothelioma development. Recent analyses have begun to elucidate the most frequent alterations that may drive mesothelioma development, uncovering disrupted genes with well-known roles in human cancer development. While these results are encouraging, they represent only a fraction of the landscape of molecular alterations that define the biology of mesothelioma. 

Future multi-omics studies will contribute to addressing the prevailing clinical challenges, particularly the identification of molecular features and subtype-specific characteristics that may be able to distinguish mesothelioma from other tumors with a high degree of sensitivity. Thus, employing these approaches in future research efforts will significantly contribute to identifying clinically actionable molecular events and subsequently result in critical improvements to patient diagnosis and prognosis.

## Figures and Tables

**Table 1:** RNA sequencing data resources on mesothelioma tissues.

Source	Number of Cases	Analysis	Platform	References
TCGA, Pan Cancer Atlas	87 MPM tissue samples	RNASeq	Illumina HiSeq 2000	[28]
EGAD00001001914	12 MPM cell lines	RNASeq	Illumina HiSeq 2000	N/A
EGAD00001001915	211 MPM samples	RNASeq	Illumina HiSeq 2000	
EGAD00001001916	207 MPM samples	Targeted Sequencing using SPET	Illumina HiSeq 2000	
International Mesothelioma Program/Brigham and Woman’s Hospital/Harvard Medical School	4 MPMs, 1 normal control, 1 lung adenocarcinoma (LAC)	Transcriptome Sequencing	Roche/454-pyrosequencing	[31,32]
Ospedale Policlinico San Martino (Genova, Italy)	26 MPM tissue samples, and 3 non-malignant pleura samples	miRNA	Human miRNA Microarray Kit Release 19.0, 8 × 60 K	[33]
Brigham and Women’s Hospital/Harvard Medical School	40 MPM samples, 5 normal pleura, 4 normal lung, 4 MPM cell lines, and 1 non-tumourigenic immortalized mesothelial cell line (SV40)	RNA	Affymetrix Human U133A	[34]
University of Vermont, College of Medicine	4 mesothelial (pleural and peritoneal) cell lines (untreated and treated with asbestos)	RNA-Seq	Illumina HiSeq1000	[35]

TCGA: The Cancer Genome Atlas; MPM: malignant pleural mesothelioma; miRNA: microRNA; RNA-Seq: RNA sequencing; Illumina HiSeq2000: Manufactured by Illumina Inc., San Diego, CA, USA; Roche 454 pyrosequencing: manufactured by F. Hoffman-La Roche AG, Basel, Switzerland; Human miRNA Microarray Kit Release 19.0, 8 × 60 K: Agilent Technologies Inc., Santa Clara, CA, USA; Affymetrix Human U133A: manufactured by Affymetrix Inc., Santa Clara, CA, USA.

**Table 2:** DNA sequencing data resources on mesothelioma tissues.

Source	Number of Cases	Analysis	Platform	Reference
TCGA Pan Cancer Atlas	87 MPM samples	DNA-Seq, Copy Number	Illumina	[28]
NYU Cancer Research	22 MPM and matched blood samples	Exome Sequencing, Copy Number	Illumina HiSeq	[36]
University of Helsinki	21 malignant mesothelioma; 26 lung adenocarcinoma; 9 normal lung/blood samples of lung adenocarcinoma	Exome Sequencing	Illumina HiSeq	[37]
University of California, San Francisco	1 MPM tissue sample and matched non-malignant tissue	Exome Sequencing	SOLiD 5500	[38]
University of California, San Francisco	78 MPM tissue samples from 69 MPM patients	Targeted Sequencing	Ion Torrent Personal Genome Machine	[38]
University of California, San Diego (Accession: PRJNA278669; ID: 278669)	7 PeM samples, 7 whole blood samples	Exome Sequencing	Illumina HiSeq 2000	N/A
EGAD00001001913	198 MPM Samples	Exome Sequencing	Illumina HiSeq 2500	N/A
EGAD00001000360	232 mesothelioma samples	Genome Sequencing, Copy Number	Illumina HiSeq 2000	N/A
EGAS00001002299/EGAS00001002298	3 pleural effusions and matched blood samples	Genome Sequencing	Illumina HiSeq X Ten/BGISEQ-500	[39]
EGAD00001001917	1 cell line (NCI-H2495)	PacBio	PacBio RS II	N/A
The International Mesothelioma Program	1 MPM sample and matched non-malignant tissue	Genome Sequencing	Illumina Genome Analyzer 2 and Roche/454-pyrosequencing	[40]
University of California, San Diego, Moores Cancer Centre	42 mesothelioma samples (pleural: *n* = 23; peritoneal: *n* = 11; pericardial: *n* = 2; subtype unknown: *n* = 6)	Genome Sequencing	Illumina HiSeq 2000	[41]
University of Turin	123 MPM tissue samples	Targeted Sequencing	Ion Torrent Personal Genome Machine	[42]

PeM: Peritoneal mesothelioma; Illumina, Illumina Genome Analyzer, and Illumina HiSeq2000: Manufactured by Illumina Inc., San Diego, CA, USA; SOLiD 5500: manufactured by Life Technologies Corporation, Carlsbad, CA, USA; Ion Torrent: manufactured by ThermoFisher Scientific, Waltham, MA, USA; BGIseq: manufactured by the Beijing Genomics Institute, Shenzhen, China; PacBio: manufactured by Pacific Biosciences Inc., Menlo Park, CA, USA.

**Table 3:** Additional resources of mesothelioma data.

Resource	Description
TCGA-MPM Project [43]	A recent analysis of 74 MPM cases with no previous treatment. Multiple high-throughput techniques were performed, including whole exome, mRNA, miRNA, ncRNA sequencing, as well as copy number analyses, DNA methylation, and reverse-phase protein array profiling. Data reveal novel extensive loss of heterozygosity in a subset of MPM cases, high expression of immune-checkpoint molecules, and a high prevalence of BAP1 alterations.
National Mesothelioma Virtual Bank [44]	Online databank of mesothelioma biosamples with associated statistics. Full access to the database allows viewing of individual patient clinical data. Tissue and blood samples can also be requested through this database.
NCBI ClinVar [45]	Database of human genetic variations that may be clinically relevant. The significance of each genetic variation to any type of disease is assessed, including malignant mesothelioma. Maintained by the National Institutes of Health (NIH), data are publicly available.

mRNA: messenger RNA; miRNA: microRNA.

**Table 4:** Studies identifying microRNAs with potential clinical applicability in the diagnosis and prognosis of malignant mesothelioma patients.

Classifier	Marker	Sample Type	Analysis	References
miR-126	Early Diagnosis/Prognosis	Serum samples	Low levels of miR-126 could differentiate MPM from healthy individuals, as well as non-small cell lung cancer patients. Low-levels also indicates worse prognosis	[98,99]
miR-29c* miR-92a miR-196b	Early Diagnosis	Plasma samples	Higher levels detected in plasma of mesothelioma patients when compared to healthy controls	[100]
miR-625-3p	Early Diagnosis	Plasma/serum samples	Higher levels detected in plasma of mesothelioma patients when compared to healthy controls. Also found upregulated in tumor specimens	[100]
miR-16 miR-17 miR-486	Early Diagnosis	Plasma and solid tissue samples	Downregulation in MPM and asbestos-exposed patients when compared to healthy controls	[101]
miR-141 miR-200a* miR-200b miR-200c miR-203 miR-205 miR-429	Diagnosis	Solid tissue samples	Downregulation of the miR-200 family of miRs is able to differentiate MPM from lung adenocarcinomas	[102,103]
miR-200c miR-193a-3p miR-192	Diagnosis	Solid tissue samples	Upregulation of miR-193a and downregulation of miR-200c and miR-192 are able to distinguish MPM from lung adenocarcinomas, adenocarcinomas from the gastrointestinal tract, renal cell carcinomas and other carcinomas	[102]
miR-103	Diagnosis	Peripheral blood samples	Downregulation is able to differentiate mesothelioma patients from asbestos-exposed controls	[104]
miR-103a-3p miR-30e-3p	Diagnosis	Plasmatic extracellular vesicles	Expression pattern is able to distinguish MPM from past asbestos-exposed patients	[105]
miR-34-b/c	Diagnosis	Serum-circulating DNA	Increased promoter DNA methylation in MPM patients when compared to benign asbestos pleurisy cases and healthy volunteers	[106]
miR-126 miR-143 miR-145 miR-652	Diagnosis	Solid tissue samples	Downregulation is capable of differentiating MPM from the corresponding non-malignant pleura	[107]
miR-132-3p	Diagnosis	Plasma samples	Downregulation of circulating miR-132 is able to differentiate mesothelioma patients from asbestos-exposed controls	[108]
miR-197-3p miR-1281 miR-32-3p	Diagnosis	Serum samples	Higher circulating levels detected in MPM patients when compared to healthy controls	[109]
miR-21 miR-126	Diagnosis	Cell lines, solid tissue and cytologic specimens	Overexpression of miR-21 and downregulation of miR-126 are able to differentiate mesothelioma from non-neoplastic samples	[110]
miR-29c*	Prognosis	Solid tissue samples and cell lines	Increased expression of miR-29c* is associated with the epithelial subtype and able to predict a better prognosis	[111]
miR-17-5p miR-21 miR-29a miR-30c miR-30e-5p miR-106a miR-143	Prognosis	Cell lines and solid tissue samples	Expression pattern is able to distinguish between different mesothelioma histopathological subtypes	[112]
miR-17-5p miR-30c	Prognosis	Cell lines and solid tissue samples	Downregulation is associated with better outcome in sarcomatoid mesothelioma patients	[112]
let-7c-5p miR-151a-5p	Prognosis	Solid tissue samples	Expression pattern correlate with overall survival and can be used to classify a risk group	[113]
miR-15b miR-16 miR-193a-3p miR-195 miR-200c	Prognosis	Solid tissue microarray	Downregulation is associated with increased expression of PD-L1 in MPM, which is a marker of poor prognosis	[114]
miR-17-5p miR-19b-3p miR-625-5p	Prognosis	Solid tissue samples	Downregulation is associated with a better prognosis in MPM patients	[115]
miR-31	Prognosis	Solid tissue samples	Downregulation is able to distinguish MPM from reactive mesothelial proliferations. However, higher levels were found in sarcomatoid samples and associate with a worse prognosis.	[116]
miR-31	Prognosis	Cell lines	Downregulation is associated with a worse prognosis and shorter time to tumor recurrence	[117]
miR-31	Prognosis	Cell lines	Upregulation is associated with an intracellular accumulation of platinum, but with a decrease intranuclear concentration promoting chemoresistance	[118]

PD-L1: Programmed death-ligand 1.

**Table 5:** Current long non-coding RNAs (lncRNAs) described to be relevant to mesothelioma.

lncRNA	Analyses	Key Findings	References
*NEAT1*	In silico analyses; Microarray; RT-qPCR	- Overall downregulation in MPM, proportion of epithelioid samples display upregulation - May be BAP1-dependent - Promotes EMT through regulation of EZH2 in other cancer types	[125,126,130,131]
*PAX8-AS1*	In silico analyses; Microarray; RT-qPCR	- Upregulated in MPM relative to benign pleura - Antisense to *PAX8* - Negative *PAX8* IHC staining can distinguish ovarian from mesothelial tumors - Co-expression network enriched in cell death and epithelium development	[126,132]
*SNHG7*	In silico analyses; Microarray; RT-qPCR	- Upregulated in MPM relative to benign pleura - Encodes small nucleolar RNAs, which aid post-translational modifications - Expression associated with hilar lymph node metastasis	[125,126]
*PVT1*	In silico analyses; NGS; In vitro siRNA knockdown	- Found in same region as myc (8q24), a region frequently gained MPM (coamplification) - Increased *PVT1* required for elevation of MYC protein levels - *PVT1* associated with cell proliferation and apoptosis inhibition	[125,128,130]
*GAS5*	In vitro and in silico analyses	- Downregulated in MPM cell lines, upregulated during growth arrest - Silencing shortened cell cycle length - May be negatively regulated by miR-21	[129,130,133]
*EGFR-AS1*	In vitro and in silico analyses	- Overexpressed in MPM - Previously described to regulate EGFR expression in liver cancer - Knockdown leads to increased sensitivity to TKIs - May regulate EMT in MPM	[130]
*PCAT6*	In silico analyses; Microarray; RT-qPCR	- Overexpressed in MPM across all subtypes, but significant in biphasic only - A prognostic and diagnostic marker in other cancers	[130]
*ZEB2-AS1*	In silico analyses	- Known regulator of ZEB2 (involved in EMT) - Expression is potentially dysregulated in MPM (not validated)	[130]
*HOTAIR*	In silico analyses	- Upregulated in sarcomatoid subset - Known oncogenic lncRNA, regulates EMT - High expression associated with poor overall survival	[130]
*MORT*	In silico analyses	- TCGA-MESO dataset shown to have strong epigenetic silencing by methylation	[130]

EMT: Epithelial-mesenchymal transition; NGS: next-generation sequencing; EGFR: epidermal growth factor receptor; TKI: tyrosine kinase inhibitor.
